# Book Reviews

**Published:** 1915-03

**Authors:** 


### BOOK REVIEWS

Books mentioned may be inspected at and ordered through this office. So far as possible, books received in any month will be reviewed in the issue of the second month following.

**Transactions of the 36th Annual Meeting of The American Laryngological Association.** Atlantic City, May 25-27, 1914.

This is a valuable collection of monographs, illustrated, and also a compilation of data regarding the association, including lists of active and past members, officers, etc. It is published by the association, whose secretary is Dr. Harmon Smith, 44 W. 49, N. Y.

**Annual Report of the Surgeon General of the Public Health Association, 1914.**

This is the usual administrative report. We note that, of 1,486,000 immigrants inspected at ports of the U. S. and Canada, 1860 were certified as idiotic, insane, epileptic and tuberculous —the reason for this grouping not being apparent—, 5,618 as being afflicted with loathsome or dangerous contagious diseases, 25,567 as having diseases affecting earning ability, 8,205 as afflicted with minor diseases and defects, total 41,250, nearly 3%. The report is of interest in its discussion of endemic and epidemic diseases and should be studied by all sanitarians.

**Transactions of The American Surgical Association,** Vol. 32, for the meeting of 1914.

The papers include treatises on surgical technic, X-Rays, surgical pathology, etc. Published by the association, Dr. Archibald MacLaren, Recorder, St. Paul, Minn.

**Text Book of Surgical Operations,** Prof. Fedor Krause, and Dr. Emil Heymann, Berlin, translated and edited by Dr. Alfred Ehrenfield, Boston. Published by the Rebman Co., New York. 6 volumes, Vol. 1 265 pages, 55 plates containing 233 illustrations in two or more colors, and 61 figures in the text, $6.00

The preliminary chapters deal with preparation for operation, anaesthesia, including the infiltration, spinal and other modern methods, asepsis, after-treatment. The work is then continued by regions, the present volume including the face and head, including the eye and orbit, ear and mastoid, nose and accessory sinuses, the trifacial nerve and its ganglia but not the cranium and cerebrum. Each operation is described in full, with allusions to clinical cases to illustrate peculiar emergencies and means of circumvention.

---

**Transaction of The American Association of Genito-Urinary Surgeons,** Vol. 9, for the 28th Annual Meeting at Stockbridge, Mass., May 15 and 16, 1914.

Most of the papers are beautifully illustrated. Published for the Association by Frederick H. Hitchcock, 105 W. 40 St., New York.

---

**Diseases of the Bronchi, Lungs and Pleura.** Dr. Frederick T. Lord of Boston; published by Lea & Febiger, Philadelphia and N. Y., 605 pages, 93 engravings and 3 colored plates, $5.00.

We would like our readers to compare this work with the respiratory diseases from almost any standard text book of even a few years ago. This part of practice had gradually been standardized so that, unless a specious effort toward originality was made, the latter was very much alike as presented by all authors and it appeared that, with minor exceptions, we could complacently regard this field of practice as a closed issue. But, with the development of Roentgenology, endoscopy and the more careful and detailed application of bacteriology, cytology, and even the collation of observation along lines well established, opportunities for advancement are still open. This is the first text book adapted to clinical use in which the matter is presented in a comprehensive way in accordance with modern developments—so far as our attention has been called to the subject in this department.

---

**Obstetrical Nursing**. A manual for nurses and students and practitioners of medicine. By Charles Sumner Bacon, Ph.B., M. D., Professor of Obstetrics, University of Illinois and the Chicago Polyclinic; Medical Director, Chicago Lying-in Hospital and Dispensary; Attending Obstetrician, University of Chicago Polyclinic, Hernotin, German and Evangelical Deaconess Hospitals. 12 mo, 355 pages, illustrated with 123 engravings. Cloth $2.00 net. Lea & Febiger, Publishers, Philadelphia and New York, 1915.

This book aims to give the nurse a fair general understanding of the nature of the disease which she or he is attending, to instruct in the management of special appliances, such as colostomy tubes, dressings, splints, etc., to teach simple tests as of the urine, and it concludes with a discussion of first aid. It is obviously a very difficult problem to establish standards of what a nurse should know and to steer between the Scylla of developing a nominally instructed but practically inexperienced practitioner of medicine and surgery and the Charybdis of an assistant ignorant of all principles and trained only in certain mechanic details. While necessarily impossible to cover all parts of a highly complicated subject perfectly and with satisfaction to all individual opinions, it seems to us that the author has solved the problem much more satisfactorily than most of those who have attempted it. We therefore advise our readers to commend this book to the attention of nurses.

---

**Infant Feeding, Its Principles and Practice.** By F. L. Wachenheim, M. D., attending physician Sydenham Hospital and Mount Sinai Dispensary, New York City. 12 mo, 340 pages. Cloth, $2.00 net. Lea & Febiger, Publishers, Philadelphia and New York, 1915.

This work is of convenient size. The author realizes the importance of a thorough scientific basis for infant feeding but also the equal or greater importance of qualifying all rules as to feeding by practical considerations, including both general and particular experience. The customary arrangement is followed.

---

**Students' Manual of Gynecology.** By John Osborn Polak, M. D., F. A. C. S., Professor of Obstetrics and Gynecology, Long Island College Hospital; Professor of Obstetrics in the Dartmouth Medical School; Gynecologist to the Jewish Hospital; Consulting Gynecologist in the Bushwick, Coney Island, Deaconess' and Williamsburg Hospitals, Brooklyn, and the Peoples' Hospital, New York; Fellow American Gynecological Society, etc. 12 mo., 414 pages, illustrated with 100 engravings and 9 colored plates. Cloth, $3.00, net. Lea & Febiger, Publishers, Philadelphia and New York, 1915.

We have previously expressed a favorable opinion as to this author's Manual of Obstetrics. This work is marked by the same conciseness and practicality. The plates showing graphically, the days of flowing in various conditions producing menorrhagia and metrorrhagia, in contrast with normal and mildly pathologic types of menstruation, are simple and instructive. The contents are logically arranged and are written with the needs of the student and general practitioner in mind.

**A Compend of Obstetrics.** Henry G. Landis, A. M., M. D., edited and revised by Wm. H. Wells, M. D., of Philadelphia. Ninth edition, 260 pages, 80 illustrations, $1.00; P. Blakiston's Son & Co., Philadelphia.

This is one of the old, familiar, brown covered quiz-compends, from which, it must be confessed, most of the elderly and middle-aged physicians now in practice, obtained the greater part of their technical education, under the somewhat rudimentary conditions of medical didactics of their student days. This edition has been thoroughly modernized and, while the quiz system has been retained, it is supplemented by tabular views and concise presentations of fact.

---

**Urinary Analysis and Diagnosis,** by Microscopic and Chemic Examinations. Louis Heitzmann, M. D., New York, 3d revised and enlarged edition, 345 pages; 131 illustrations, mostly original, $3.00. Wm. Wood & Co., New York.

The author has made no specious pretense at originality by departing from the plan of arrangement now quite well established as standard so that no difficulty will arise from using this work in place of older books. At the same time, there has been considerable new matter added. The illustrations, both of histologic construction of the urinary organs and of microscopic findings in the urine are shown much larger than is customary. This has a great advantage from the standpoint of the learner or of anyone with defective vision. It combines the advantages of actual pictures and of diagrams. Yet it has the draw-back of establishing mental pictures on a far larger scale than those obtained from actual clinical work and, as no greater definition is secured, of leading the inexperienced to look for coarser and plainer appearances than will actually be found. An analogous balance of advantage and disadvantage occurs in some very brilliantly and distinctly colored plates in certain works on haematology. While there is much to be said on either side of the question, we rather incline to the opinion that the text book should reproduce, as nearly as possible, the difficulties of visual interpretation which will actually be found in practice. With this debatable point in mind, the work is excellent, especially in adopting a standard of thorough investigation, yet without exceeding the ability and resources of the physician.

---

**A Practical Text-Book of Infection, Immunity and Specific Therapy** with special reference to immunologic technic. By John A. Kolmer, M. D., Dr. P. H., Instructor of Experimental

Pathology University of Pennsylvania, with an introduction by Allen J. Smith, M. D., Professor of Pathology, University of Pennsylvania. Octavo of 899 pages with 143 original illustrations, 43 in colors. Philadelphia and London: W. B. Saunders Company, 1915. Cloth, $6.00 net, half Morocco, $7.50 net.

We regret very much that the term immunologic, instead of amynologic is to be more firmly fixed in the medical vocabulary by its acceptance by the present author, whose work is bound to be a landmark in medical science. Contrary to what might at first thought be considered the logical order, the first part of the book discusses the technic of making the necessary glassware, of obtaining animal and human blood, of inoculating animals, of preserving serums, while part 2 deals with infection and part 3 with the principles of immunity, including much technic. This arrangement is, however, strictly logical, since it affords the opportunity for developing the theoretic considerations by object lessons while, even for those who do not contemplate laboratory study, it renders the discussion more tangible. Part 4 deals with applied immunity in prophylaxis, diagnosis and treatment and it includes a chapter on chemotherapy mainly devoted to salvarsan. Part 5 is a schedule of 60 exercises, covering the principal points previously discussed. Excepting for the last part, the work is subdivided into chapters, numbered continuously and each covering a special topic. The chapter on the Wasserman reaction and its modifications covers over 70 pages—nearly as much as the entire first part of the work and is elaborately illustrated in colors. It would seem that this chapter ought to eliminate a good share of the inconsistent results reported in the diagnosis of syphilis. The next chapter to this, of considerably less size, includes the other complement fixation reactions. We recommend this work not only to laboratory workers but to the profession generally as it is important for all to understand the scope and the limitations of immunity and to realize what may be done in the laboratory to elucidate problems of infection.

---

**Diagnostic and Therapeutic Technic.** A Manual of Practical Procedures Employed in Diagnosis and Treatment. By Albert S. Morrow, M. D., Clinical Professor of Surgery, New York Polyclinic. Second edition, thoroughly revised. Octavo of 834 pages, with 860 illustrations. Philadelphia and London, 1915. Cloth, $5.00 net; half Morocco $6.50 net.

It is rather difficult to define accurately the scope of this book. Perhaps we may say that it deals with the procedures which the internist is required to use in the more elaborate methods of diagnosis and treatment and which require some degree of surgical skill. General and local anaesthesia, including infiltration methods, Bier's venous method, spinal and sacral anaesthesia; sphygomanometry, transfusion of blood, acupuncture, venesection, cupping, etc., hypodermic and intramuscular injections of drugs, including salvarsan, injection treatment of neuralgia, collection of various pathologic material and excretions, exploratory puncture, endoscopy and a good deal of the technic of the special sense, genito-urinary and abdominal organs, are comprised in this work. Granted that a man has acquired the usual medical education, is fairly well trained in ordinary physical diagnosis and therapeutics, it is just the book that he needs in order to equip himself for really thorough general practice and to follow intelligently specialists in various lines, to the degree necessary if one is not content to refer every patient who presents indications calling for more than the average skill, or even if, with such reference he wishes to regard himself as an intelligent collaborator.

---

**International Clinic Week.** International Surgical Congress, 1914.

This is a hundred-page memorial of the work at the New York Polyclinic during the International Surgical Congress in April, 1914. It is copiously illustrated and is a valuable record of surgical progress as noted at the clinics.

---

**Practical Medicine Series,** edited by Charles L. Mix, A. M., M. D., and Roger T. Vaughn, Ph. B., M. D., 10 volumes a year, $10.

Vol. 7, Obstetrics, edited by Joseph B. De Lee, A. M., M. D., and Herbert M. Stowe, M. D., 233 pages, illustrated, price separately $1.35.

Vol. 8, Therapeutics, Preventive Medicine, Climatology, edited by George F. Butler, Ph. B., A. M., M. D., Henry B. Favill, A. B., M. D., and Norman Bridge, A. M., M. D. 384 pages, illustrated, price separately $1.50.

Vol. 10, Nervous and Mental Diseases, 230 pages, illustrated, price separately $1.35, edited by Hugh T. Patrick, M. D., and Peter Bassoe, M. D.

We have already reviewed other volumes of the 1914 series. This is the most practical annual index medicus published, the parts appearing at different dates so as to be available for reference at convenient intervals and without unnecessary delay, each volume of each yearly series taking up the review of periodic literature where the corresponding volume of the previous series left off. Either the entire series or any part desired may be ordered. The Year Book Publishers, 327 South La Salle St., Chicago.

---

**The General Education Board.** An Account of Its Activities, 1902 to 1914. Published by the Board, 240 pages, 32 full page illustrations and 31 maps.

This is a statement of the organization and operation of the Board, its resources and expenditures and a survey of agricultural education especially for boys and girls, high school and college education, medical, rural and negro education. The map of the distribution of the 687 institutions which confer academic degrees is especially interesting as correcting some preconceived notions as to longitude. Excepting Boston, New York, Philadelphia, Washington and Baltimore and their immediate vicinities, where population and natural tendencies to establish educational centers combine, the map is most heavily dotted in what may be termed, paradoxically, the eastern western and the northern southern states. Various other maps show the percentage of students drawn from 50 and 100 mile circles, some colleges having as high as 93 per cent. of their students derived from within a radius of 100 miles, others only a little over 20 per cent.—the variation depending, of course, on general prestige, specialization of course, and density of population of the natural tributary area. The book is not, however, essentially statistic but discusses general principles and problems of education in a way that is both instructive and entertaining. While the work of the Board has been criticized as an unauthorized interference and as liable to usurp power by reason of its financial strength, it must be admitted that its influence has been, on the whole, most salutary and we think that the highest praise should be awarded to the general principle of voluntary taxation in the interests of the country, exemplified by the foundation.

---

**Some Important Memoranda For the Busy Physician.** Pamphlet issued by the Fellows Medical Manufacturing Co., Ltd., N. Y. Obtainable by the profession on request.

This is a collection of data, mainly regarding diagnosis, from medical journals during the past year, the authorities being cited in a bibliographic list. Twilight sleep, Osler's spots, various signs of nearly pathognomonic value, formulae for iodine and other solutions, stains, and a variety of diagnostic tests are discussed. Among the last are Abderhalden's, Babinski's,

Boas's, Einhorn's thread, Garrod's for gout, Gluzinski's for gastric ulcer, several tests for occult blood, etc. Able editing is shown in the selection and in the condensation of lengthy reports and the result is a great saving of time for reference.

---

**A Text Book of Diseases of The Nose and Throat.** By D. Braden Kyle, A. M., M. D., Professor of Laryngology and Rhinology, Jefferson Medical College, Philadelphia. Fifth edition, thoroughly revised and enlarged. Octavo of 856 pages with 272 illustrations, 27 of them in colors. Philadelphia and London; W. B. Saunders Company, 1914. Cloth, $4.50 net.

This is a systematic text book of the highest order, tested and approved by long use. The chapters on bronchoscopy and speech defects deserve special mention, not because they are of higher order than the rest of the book but because they might not perhaps be remembered as part of the domain of Nose and Throat work.

---

**Differential Diagnosis.** Vol. II. Presented through an Analysis of 317 cases. By Richard C. Cabot, M. D., Assistant Professor of Clinical Medicine, Harvard Medical School. Octavo of 708 pages 254 illustrations. Philadelphia and London: W. B. Saunders Company, 1914. Cloth, $5.50; Half Morocco, $7.00.

This book, while designated as Vol. II, continuing the general subject of diagnosis according to prominent symptoms and signs, is independent so far as practical use is concerned. Vol. I dealt with pain. The author and publisher are silent as to subsequent volumes but we trust that they will be forthcoming. This work includes Abdominal and other Tumors—with mention of pregnancy—Vertigo, Diarrhoea, Dyspepsia, Haematemesis, Glands, Blood in the Stools, Swelling of the Face, Haemoptysis, Oedema, Frequent Micturition and Polyuria, Fainting, Hoarseness, Pallor, Swelling of the Arm, Delirium, Palpitation and Arrhymia, Tremor, Asciter and Abdominal Enlargement. The lack of attempt at an impossible tabular classification is good evidence of the practical nature of the work. Under each heading, a number of types are represented by carefully studied clinical cases. In this way, the illustrative value of the clinic is combined with the systematic instruction of the didactic lecture and the result is a highly satisfactory work, both scientific and practical.

**Double Flowering in Plant and in Man.** Advance pages from a book by Robert T. Morris, M. D., entitled "Tomorrow's Topics" to be published in May by Doubleday, Page & Company, New York.

Until the advent of Bose, with his electro-physiological experiments, plant physiologists had not taken up the study of protoplasmic phenomena along the lines which had already given such high development of our knowledge of animal physiology. We now know that the relation between stimulus and response forms a gauge of the physiological condition of the plant organism. It is only very recently that we could assume that an overcultivated rose would become nervous and excitable for the same reason that an over-cultivated animal becomes nervous and excitable.

The behavior of a plant or of an animal depends largely upon the degree of microbe influence upon its four-block (Helium hypothesis) system of cell protoplasm. Good microbes lend a hand in assisting energy in its task with helium of building up plants and animals. Bad microbes interfere. Both kinds are always present, incessantly taking part in construction or demolition of protoplasm. When the gardener cultivates ground, he wishes to assist his plants through the agency of good microbes which develop as a result of his efforts. That is all that cultivation means. The gardener overlooks the fact that his efforts at the same time favor increase of bad microbes, and heighten the effort of the plant to resist their activities.

Under conditions of over-stimulation of growth in plants during the process of higher cultivation, it is a fact that microbes, although unseen, increase tremendously under the same conditions. We must always remember that cultivation for plants means nothing but giving microbes a better chance for growing. It brings a new responsibility upon the protecting soma cells of plants,—they have too much to do. Soma cells are obliged to neglect the germ cells or else to call them over into action as allies. This gives one explanation for the doubling of the flower. Some of the germ cells ally themselves with soma cells and make petals out of stamens. Finally not enough germ cells are left for developing ovules or pollen sufficient for construction of gametes. High cultivation of plants serves man's purpose by developing certain characteristics of root and fruit which he desires, but man forgets that he is not helping nature through his selfish action (selfish not used in sentimental meaning, but only in a comparative way). High degree of cultivation of animals, including man, has the same general effect as in plants. Soma cells are called upon to wage warfare against microbes, which increase tremendously under conditions of town life. Germ cells are neglected under conditions of town life. Germ cells are neglected, or called over to become allies of the soma cells. Neglected germ cells then go to skirmishing or to morbidly protecting, instead of remaining in reserve for proper natural use. They may set the mind of man at constructing the selling part of modern realistic literature, or at class teaching of sexual questions in the schools. They may cause perversion of function, followed by abnormal excess or abnormal absence of the natural impulse belonging to natural structures.

The plant or animal then, under stimulation of a cultivation which at the same time cultivates microbes in excess, must suffer injury through the increased efficiency of its enemies. If we realize that such increase in efficiency of enemies is under way, the situation will be met as well as possible by man. This has not been understood. It is to be one of the privileges of life in the coming part of the present century for men to get into an understanding of the warfare between higher organism and microbe, and to conduct culture that the enemy will at least be met on fair terms. At the present time, the enemy is not being met on even terms, as we may note in the object lesson of rapid decadence of cultivated plants and nations.

When cultivation has finally carried a certain plant to the point where its stamens have all gone to form petals, it has no more good sex cells left for continuing its race. Very much the same process occurs among animals. In addition, the protoplasm of any species is wound up by nature, and given a certain time to run, before becoming senescent. Senescence of protoplasm is hastened by processes incidental to cultivation. The logical end of culture then is elimination of the race among plants and among animals. The superman is the man who is bringing his family lineage to a close.

A double rose among plants, and a genius among men, represent the same thing.

Some doubling flowers possess great physical vigor, however, and carry a sufficient number of sex cells,—the American beauty rose for instance.

Some genii possess great physical vigor, and are enabled to have strong progeny. Henri Poincare for instance.

Not all plants of a family begin to double at the same time, when subjected to similar conditions.

The double rose and the genius, however, have entered the sensitive vulnerable group in which an excessive degree of care is required for maintaining their equilibrium as organisms.

Most of the plants with double flowers are so vulnerable to microbic influence that they readily succumb or produce freaks in progeny,—if they have any. Most of the geniuses are so vulnerable to microbic influences that they succumb easily, or produce freaks in progeny,—if they have any. A double rose may have progeny which, if left to struggle, will either fail quickly or will revert to a simple type. The progeny of a genius when left to struggle will quickly succumb or will have a tendency to revert toward a primitive type of man.

The progeny of a double flower, or of a genius then, will have to start off again under conditions of competition. This may allow the development finally, in later generations, of more doubling flowers or of more geniuses.

In such way as this nature sets limitations, prohibiting too rapid development of species of plants or of animals, in order to conserve the protoplasm of a species against premature senescence.

Nature makes progress through development steadily of the mean type,—and proper aid on the part of man consists in making selection of the best mean types for purposes of propagation. The double rose crossed with another double rose does not altogether pleasure nature, and she finally pulls the whole lot of progeny to pieces,—if any progeny is allowed.

We ask why nature has such definite plans. The answer lies with infinity,—out of our reach. We are only allowed to note an object lesson like that of the oak tree. Nature wishes the oak tree in her landscape plans. If a fine oak then is wanted in the landscape plans, why is it not allowed to grow to a height of eighty feet in one day, and save time? That would be the business man's way of doing things.

Nature says: "Ha! Ha! this is my game, not yours, I want other things to grow also. The oak must struggle with its competitors when it is little, and with microbes all of the time. If it has patience and good nature, and at the same time is a successful fighter, it is one of the things I want. If it cannot win, I don't want it, because I am playing a game which in the end will consist of winning players only. I allow men to vainly think the world is theirs in order to give them that degree of encouraging personal interest which will keep them playing at my game."

